# Discovery and control of culturable and viable but non-culturable cells of a distinctive *Lactobacillus harbinensis* strain from spoiled beer

**DOI:** 10.1038/s41598-018-28949-y

**Published:** 2018-07-30

**Authors:** Junyan Liu, Yang Deng, Lin Li, Bing Li, Yanyan Li, Shishui Zhou, Mark E. Shirtliff, Zhenbo Xu, Brian M. Peters

**Affiliations:** 1 School of Food Science and Engineering, South China University of Technology, Guangzhou, 510640 China; 2 School of Chemical Engineering and Energy Technology, Dongguan University of Technology, Dongguan, 523808 China; 3 Department of Clinical Pharmacy, College of Pharmacy, University of Tennessee Health Science Center, Memphis, TN 38163 USA; 4 College of Food Science and Engineering, Qingdao Agricultural University, Qingdao, 266109 P.R. China; 5 Guangdong Province Key Laboratory for Green Processing of Natural Products and Product Safety, Guangzhou, 510640 China; 6 Department of Cell Biology, Harvard Medical School, Boston, MA 02115 USA; 7 School of Bioscience and Bioengineering, South China University of Technology, Guangzhou, 510006 China; 8 Department of Microbial Pathogenesis, School of Dentistry, University of Maryland, Baltimore MD, MA 21201 USA

## Abstract

Occasional beer spoilage incidents caused by false-negative isolation of lactic acid bacteria (LAB) in the viable but non-culturable (VBNC) state, result in significant profit loss and pose a major concern in the brewing industry. In this study, both culturable and VBNC cells of an individual *Lactobacillus harbinensis* strain BM-LH14723 were identified in one spoiled beer sample by genome sequencing, with the induction and resuscitation of VBNC state for this strain further investigated. Formation of the VBNC state was triggered by low-temperature storage in beer (175 ± 1.4 days) and beer subculturing (25 ± 0.8 subcultures), respectively, and identified by both traditional staining method and PMA-PCR. Resuscitated cells from the VBNC state were obtained by addition of catalase rather than temperature upshift, changing medium concentration, and adding other chemicals, and both VBNC and resuscitated cells retained similar beer-spoilage capability as exponentially growing cells. In addition to the first identification of both culturable and VBNC cells of an individual *L. harbinensis* strain from spoiled beer, this study also for the first time reported the VBNC induction and resuscitation, as well as verification of beer-spoilage capability of VBNC and resuscitated cells for the *L. harbinensis* strain. Genes in association with VBNC state were also identified by the first genome sequencing of beer spoilage *L. harbinensis*. The results derived from this study suggested the contamination and spoilage of beer products by VBNC and resuscitated *L. harbinensis* strain BM-LH14723.

## Introduction

First reported in 1982, Viable but nonculturable (VBNC) state has been well established and documented to be a survival strategy of nonsporeforming bacteria in response to natural stress, such as starvation, extreme temperature, elevated osmotic pressure, oxygen concentration, or exposure to visible light^1,2^. Bacteria in VBNC state have been considered to be a significant issue in public health and food safety, as on one side they fail to grow and form colonies on the routine bacteriological media, but remain alive and retain metabolic activity, and on the other they could regain culturability after resuscitation^3^.

Beer is a universally popular beverage and has a large consumption market. Despite high microbiological stability, beer spoilage incidents caused by microorganisms, such as lactic acid bacteria (LAB), have occasionally been reported due to false negative routine detection for microorganism^4,5^. However, none of beer spoilage caused by VBNC bacteria has been verified and confirmed.0

*Lactobacillus harbinensis*, a newly identified beer-spoilage LAB, produces lactic acid as end product, acetic acid and diacetyl as metabolic byproducts during carbohydrate fermentation which significantly and undesirably influence the flavors of beer. Occasional beer spoilage incidents due to false-negative detection of causative microorganisms, result in significantly profit loss and have been considered to be a leading problem in the beer brewing industry^6–8^. Failure to detected these spoilage agents, are partially due to the use of traditional culture-based techniques, which are unable to reliably detect the VBNC microorganisms commonly found under high levels of microbial stress^5,8–11^.

As occasionally reported, finished beer that previously passed random microorganism detection by “golden standard” routine culturing, are found spoiled after shelf storage. In this study, both culturable and VBNC cells of an individual *L. harbinensis* strain BM-LH14723 were identified in one spoiled beer sample, with the induction, resuscitation and characteristics of the VBNC state further defined. This study represents the first report of beer spoilage by both culturable and VBNC *Lactobacillus* cells, and demonstration of *L. harbinensis* entry into and resuscitation from the VBNC state. The first genome sequence of *L. harbinensis* is also reported.

## Results

### Identification of *L. harbinensis* strain in spoiled beer sample

**Figure 1:**
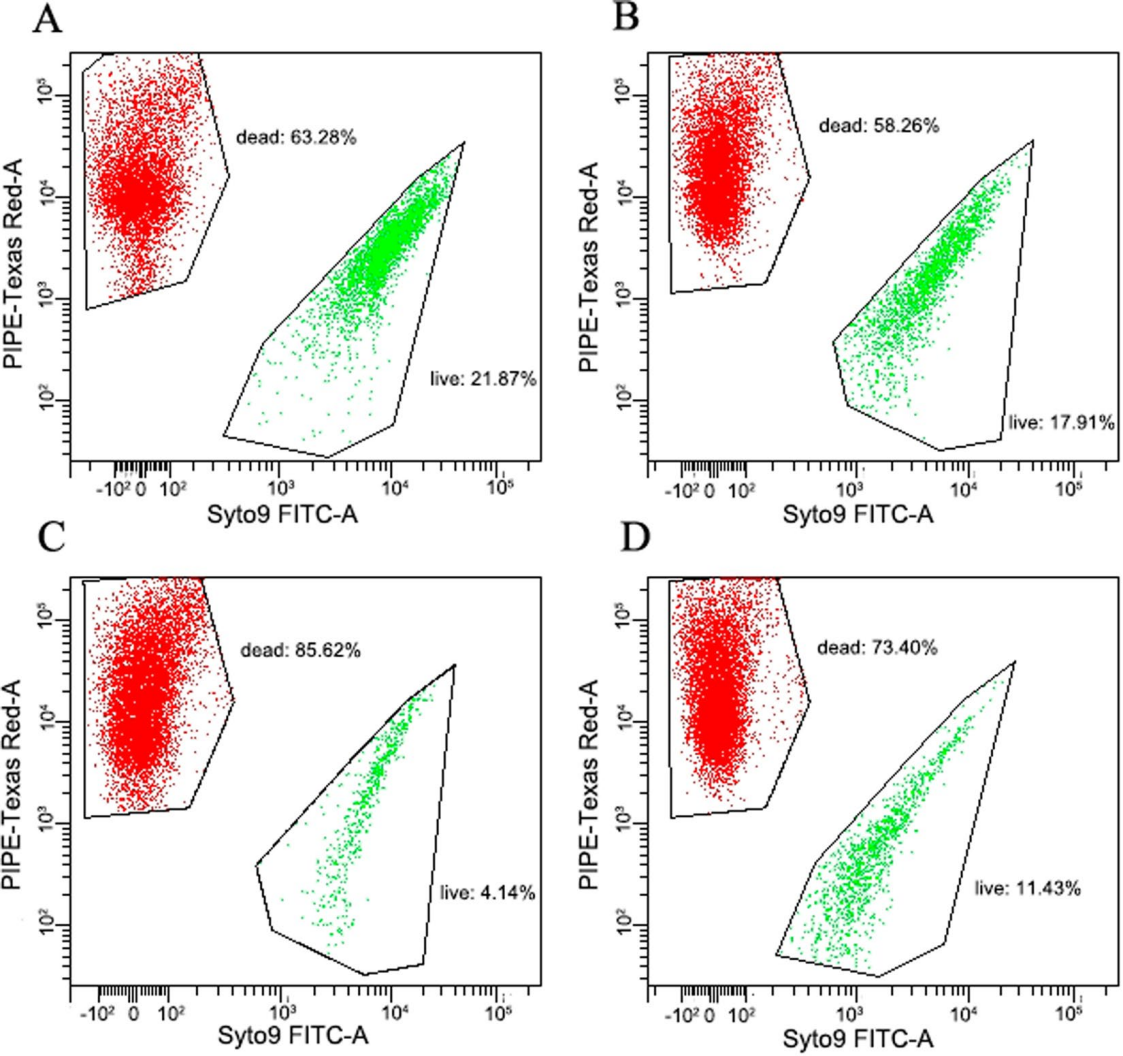
Flow cytometry analysis of bacterial cells in the spoiled beer sample (**A** and **B**) and VBNC cells induced by low temperature storage in beer (**C**) and continuous passage in beer (**D**). The live cells (green fluorescence, SYTO9) and dead cells (red fluorescence, PI) are viewed simultaneously by appropriate excitation and emission spectra.

According to MRS agar growth, AODC and Live/Dead BacLight bacterial viability kit methodologies with fluorescent microscopy and flow cytometer (Fig. 1), the difference between culturable and viable cell number was approximately 4 × 10^2^ cells/mL, demonstrating the presence of VBNC cells in the specific spoiled beer sample acquired in Guangzhou of South China in 2014. After 16*S rRNA* gene sequencing, both bacterial cells in the spoiled beer sample and the isolated *L. harbinensis* cells were further investigated by genomic sequencing. After assembly and alignment, the genomic sequences showed >99% similarity, highly suggesting both the culturable and VBNC cells in the spoiled beer sample were one distinctive *L. harbinensis* strain (named BM-LH14723).

### General genome features

**Figure 2:**
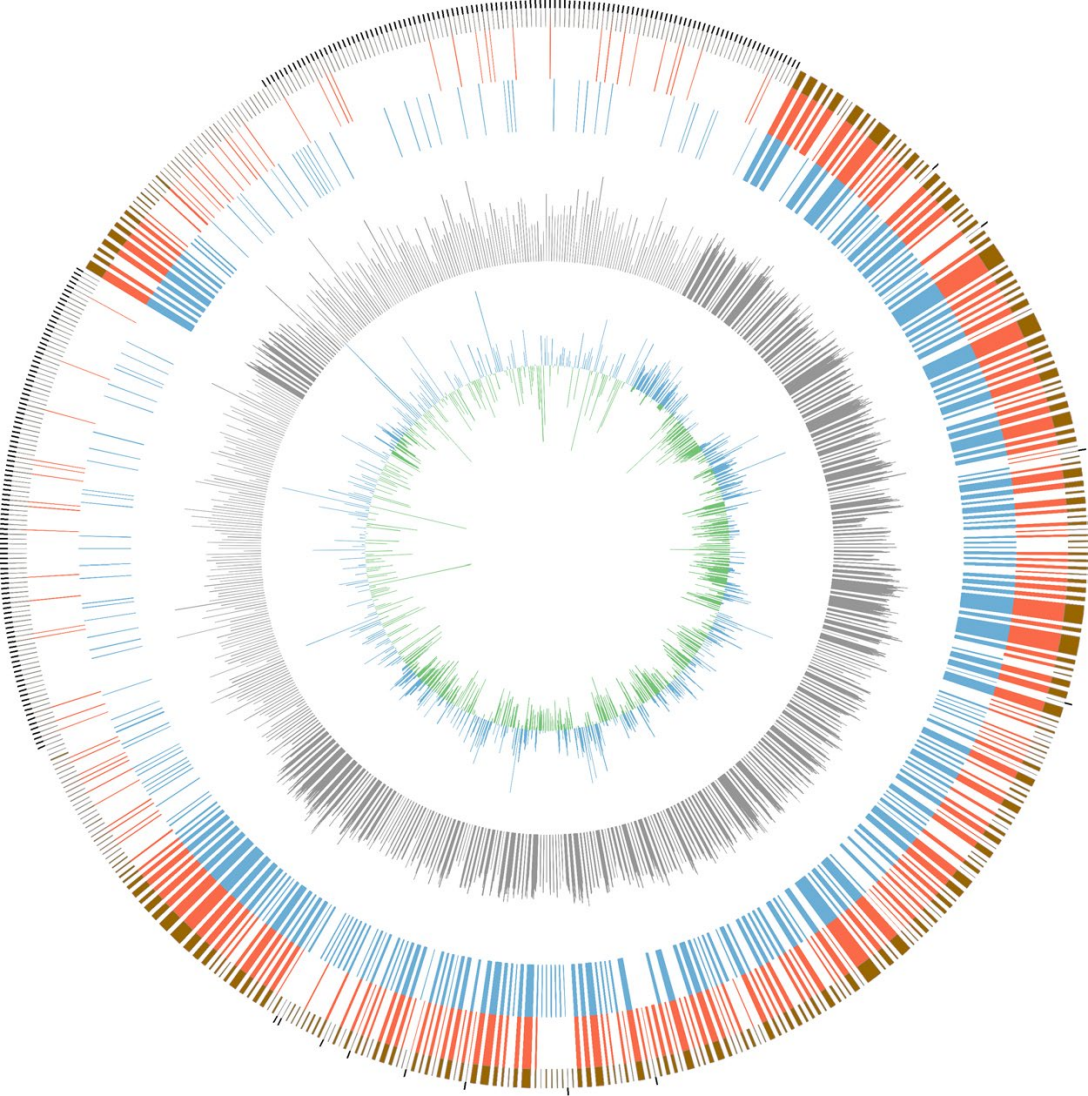
The genomic information of L. harbinensis strain BM-LH14723. The circle from outermost to innermost illustrates scaffold sequences, genes in plus strand, gene in minus strand, GC content, low GC content sequences, and high GC content sequences, respectively.

**Figure 3:**
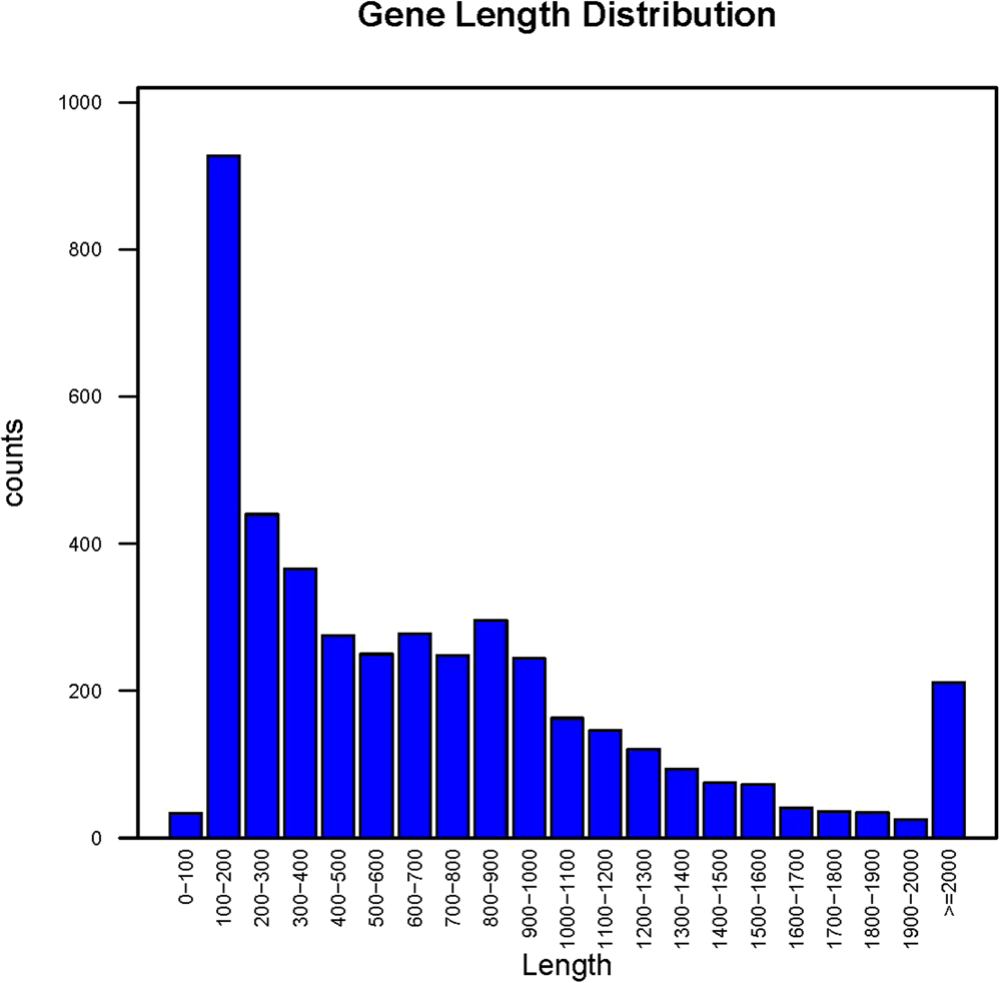
Gene length distribution of the L. harbinensis strain BM-LH14723.

**Figure 4:**
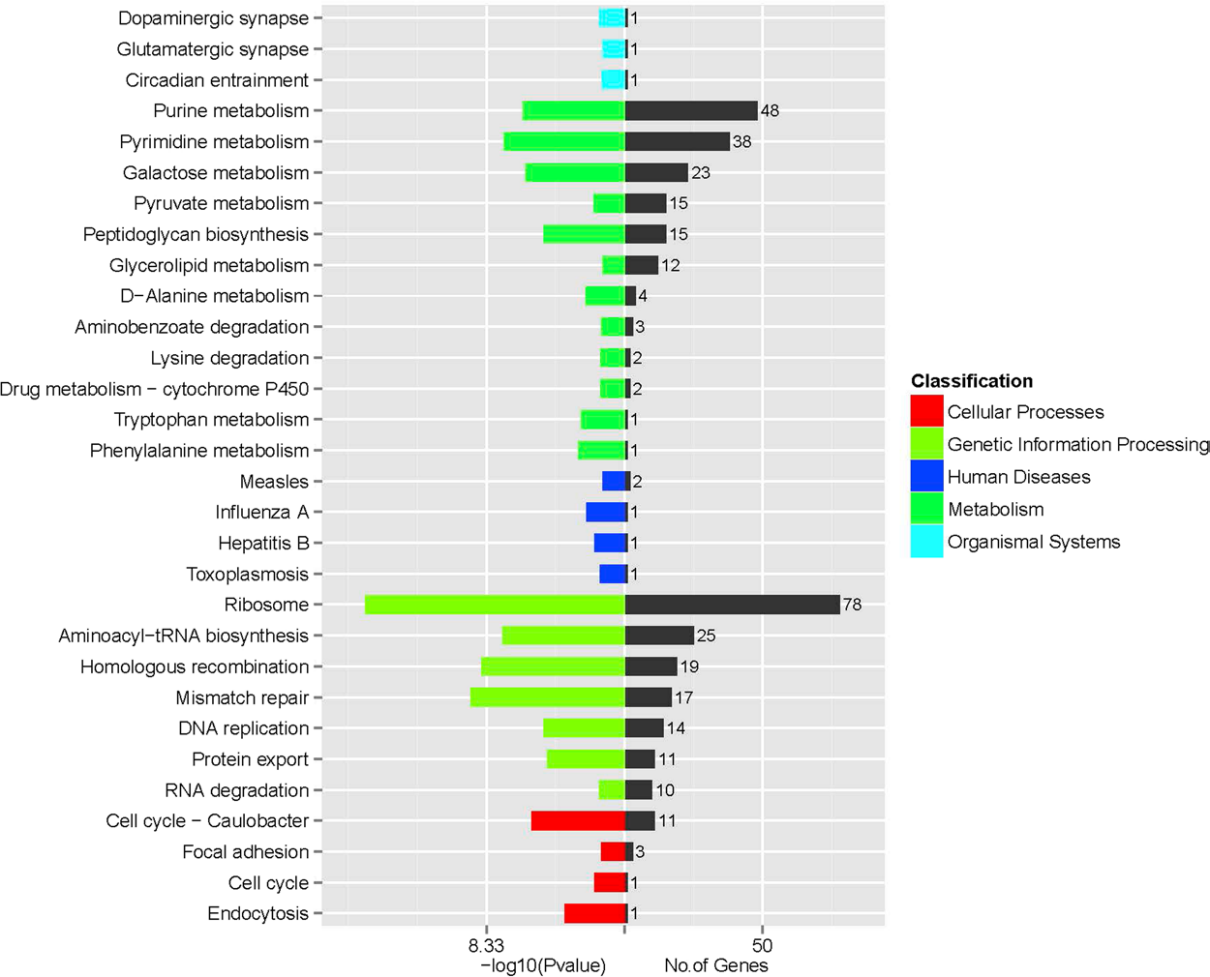
KEGG pathways distribution of the genes in the L. harbinensis strain BM-LH14723.

**Figure 5:**
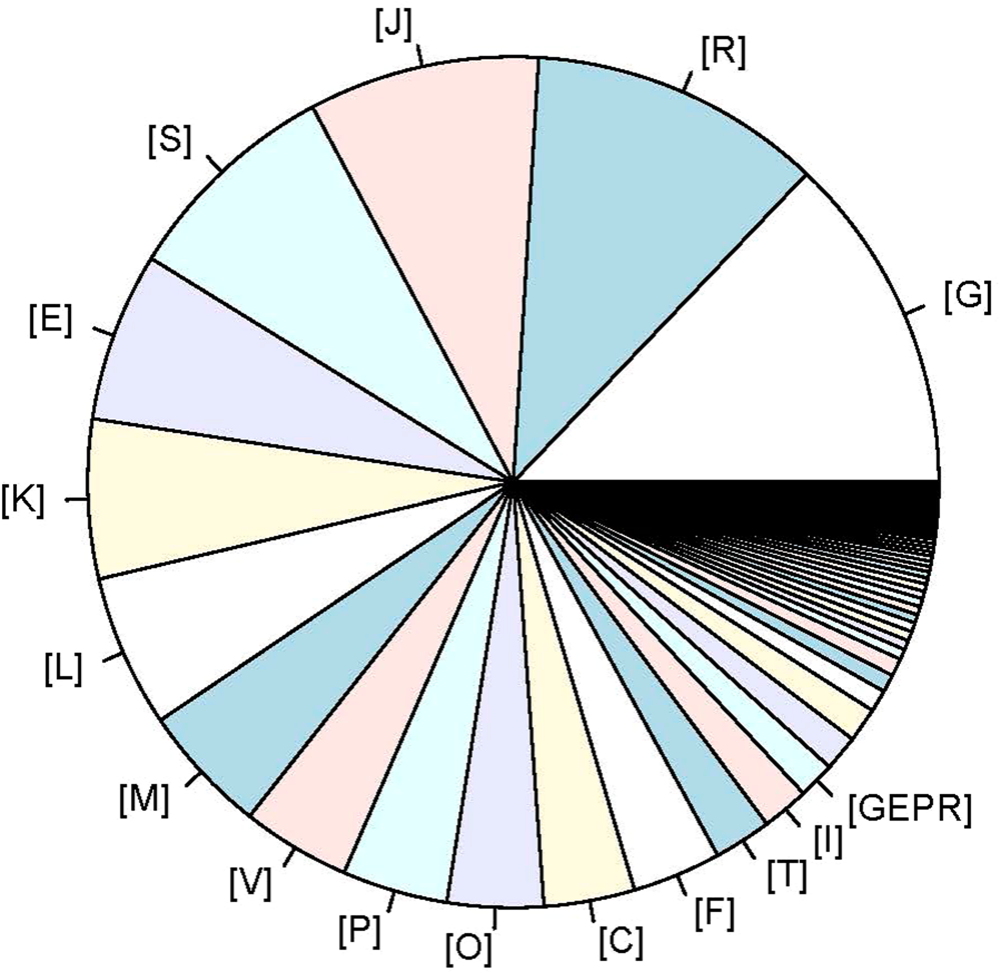
COG categories distribution of the genes in L. harbinensis strain BM-LH14723. [G]: Carbohydrate transport and metabolism; [R]: General function prediction only; [J]: Translation, ribosomal structure and biogenesis; [S]: Function unknown; [E]: Amino acid transport and metabolism; [K]: Transcription; [L]: Replication, recombination and repair; [M]: Cell wall/membrane/envelope biogenesis; [V]: Defense mechanisms; [P]: Inorganic ion transport and metabolism; [O]: Posttranslational modification, protein turnover, chaperones; [C]: Energy production and conversion; [F]: Nucleotide transport and metabolism; [T]: Signal transduction mechanisms; [I]: Lipid transport and metabolism; [GEPR]: Permeases of the major facilitator superfamily.

**Figure 6:**
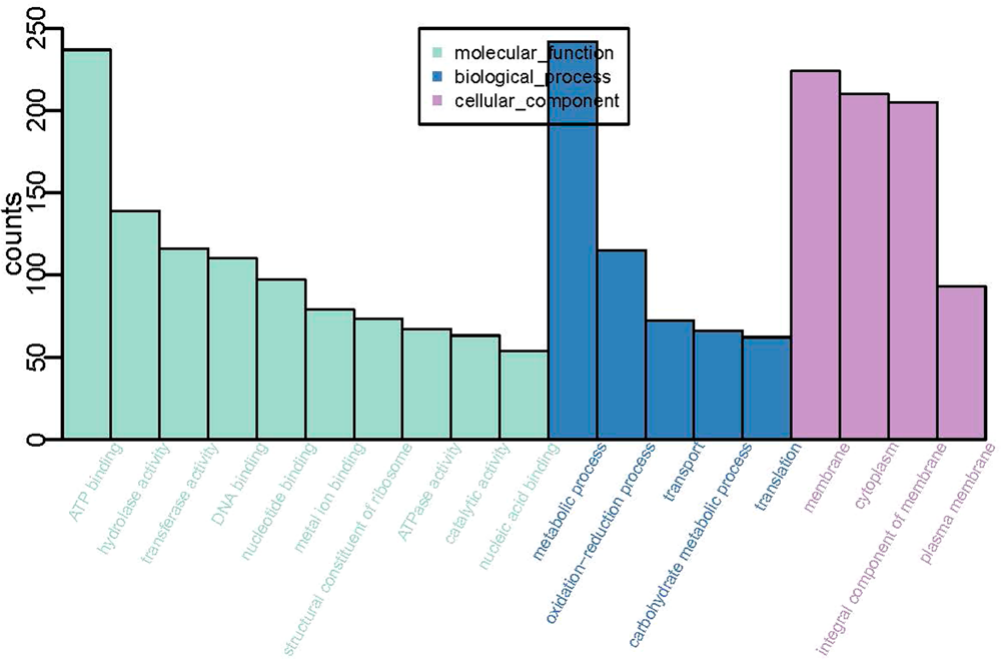
Enriched GO terms distribution of the genes in L. harbinensis strain BM-LH14723.

The genome size of the *L. harbinensis* strain BM-LH14723 is 3,017,769 bp with a coverage of 99.89%, and the average G + C content is 53.36% (Fig. 2, GenBank accession number: LTDZ00000000). A total of 4,378 genes (including 3 *rRNA* and 14 *tRNA)* most of which ranged from 100 bp to 1000 bp were acquired (Fig. 3) and annotated against Kyoto Encyclopedia of Genes and Genomes (KEGG) pathway (Table S1), Clusters of Orthologous Groups of proteins (COG) (Table S2), Gene Ontology (GO) (Table S3), and NCBI-NR databases (Table S4). The KEGG pathways were classified into 5 classes and most of the significantly enriched KEGG pathways were genetic information processing including “Ribosome”, “Mismatch repair”, “Homologous recombination”, “Aminoacyl-tRNA biosynthesis”, “Pyrimidine metabolism”, etc (Fig. 4). Pathways classified into human diseases including “Measles”, “Influenza A”, “Hepatitis B”, and “Toxoplasmosis” were also acquired by *L. harbinensis* strain BM-LH14723 (Fig. 4). For the COG categories acquired by *L. harbinensis* strain BM-LH14723 (Fig. 5), “[G] Carbohydrate transport and metabolism”, “[R] General function prediction only”, and “[J] Translation, ribosomal structure and biogenesis”, etc. were significantly enriched. As to the enriched GO terms (Fig. 6), “ATP binding”, “hydrolase activity”, “transferase activity” etc. in molecular function, “metabolic process”, “oxidation-reduction process”, “transport”, etc. in biological process, and “membrane”, “cytoplasm”, “integral component of membrane”, etc. in cellular component were identified.

**Figure 7:**
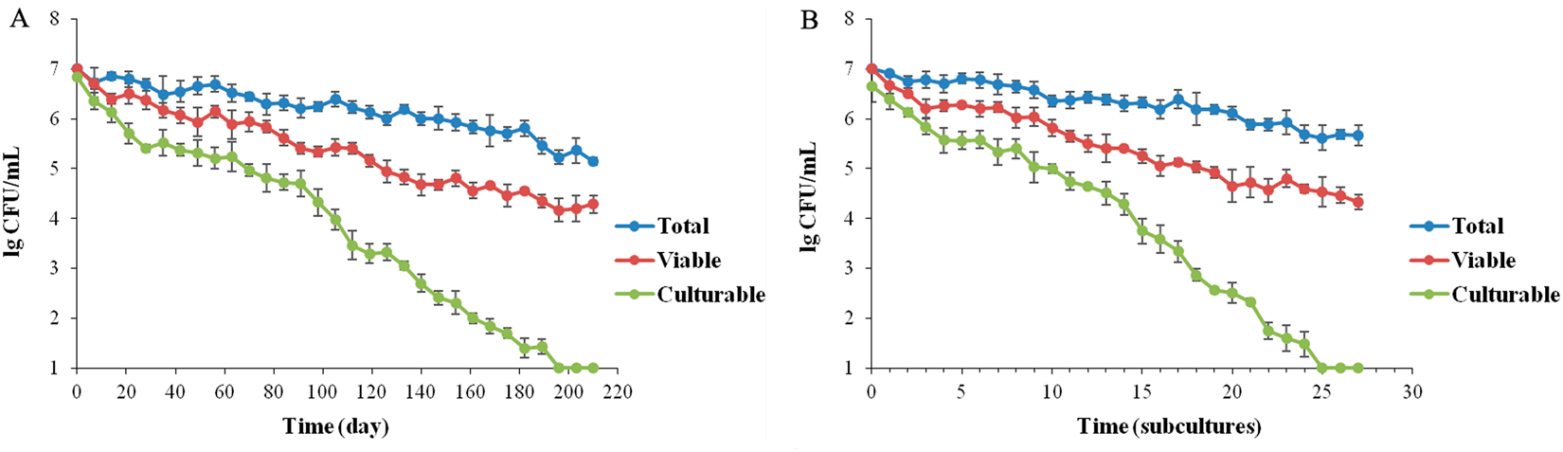
Entry of L. harbinensis BM-LH14723 into the VBNC state upon low-temperature storage (0 °C) in beer (**A**) or continuous passage in beer (**B**), respectively.

### Formation and identification of VBNC state

Approximately 10^4^ cells/mL VBNC cells of *L. harbinensis* strain BM-LH14723, a newly identified beer spoilage strain, were obtained under low-temperature storage in beer (175 ± 1.4 days, Figs 1C and 7A) and beer subculturing (25 ± 0.8 subcultures, Figs 1D and 7B), respectively. Aside from traditional plate counting coupled with AODC and Live/Dead BacLight bacterial viability kit methodologies, PMA-PCR had been proposed to identified VBNC cells^12^. As PMA penetrates only into dead bacterial cells with compromised membrane integrity, but not into live cells with intact cell membranes, PMA treatment of cultures with both viable and dead cells results in selective removal of DNA from dead cells^12^. The VBNC state of *L. harbinensis* BM-LH14723 induced by conditions of low-temperature storage in beer and beer subculturing were also approved by PMA-PCR amplification of 16 *S rRNA* gene in nonculturable cells (data not shown) to identify the presence of microbes.

### Resuscitation of VBNC cells

Proving that true resuscitation of cells from the VBNC state occurs has been problematic among bacteria^13^. The most common VBNC-resuscitating factor for low-temperature induced VBNC cells is a simple temperature upshift^14–17^. However, culturable cells were not obtained when the VBNC *L. harbinensis* cells induced by either protocols were subjected to temperature upshift (data not shown), highly suggesting cold stress is not essential for the VBNC state formation of *L. harbinensis*. An advanced beer-spoiler detection (ABD) medium with the supplementation of a small amount of MRS medium had been proposed and considered to be an effective tool for comprehensive detection of beer-spoilage LAB in breweries^18^. ABD medium was reported to allow the selective growth of beer-spoilage LAB^18^. Thus, different concentrations of media were attempted to regain the culturability of VBNC *L. harbinensis* cells. Unfortunately, change medium concentration was not an effective way to resuscitate VBNC state. Considering the oxidative stress the bacterial cells encountered during the formation of the VBNC state under cold treatment and harsh beer condition, relieving the oxidative stress could be a good way to resuscitate the VBNC state. In this study, VBNC *L. harbinensis* cells regained culturability on media containing all concentrations of catalase tested. Importantly, heat denaturation of catalase inhibited the resuscitation process. The results demonstrated that the addition of catalase is an effective method for the resuscitation of VBNC *L. harbinensis* cells.

### Beer-spoilage ability

With viable cells obtained for all 3 groups, culturable cells were only detected in beers inoculated with exponentially growing and resuscitated cells excluding that inoculated with VBNC cells, suggesting consistency of VBNC cells during the 30 days. With none of turbidity obtained in the negative control, exponentially growing, VBNC and resuscitated cells were capable of maintaining in beer within approximately 10 days and cause further turbidity, possibly suggesting the maintenance of beer-spoilage ability by *L. harbinensis* strain BM-LH14723 under both the VBNC and resuscitated state. During the 30 days, viable cells were detected in beer samples inoculated with the 3 groups of cells, while culturable cells were only identified in beer samples inoculated with exponentially growing and resuscitated cells. After 30 days of incubation, a high concentration of lactic acid and acetic acid which may eventually lead to beer acidification and high level of diacetyl which imparts a “buttery” off flavors were detected in beer samples inoculated with the exponentially growing, VBNC, and resuscitated state of *L. harbinensis* strain BM-LH14723 (Table 1).

**Table 1:** Result of beer spoilage ability determination test.

Strain	State	Turbidity	Diacetyl (mg/L)	Lactic acid (mg/L)	Acetic acid (mg/L)
*L. harbinensis* BM-LH14723	Exponentially growing	+	0.185	186.27	178.62
	VBNC	+	0.107	190.21	167.92
	Resuscitated	+	0.193	184.82	182.17

+Positive.

## Discussion

As occasionally reported, finished beer previously passed random microorganism detection by routine culturing method, are found spoiled after shelf storage. In the current study, both culturable and VBNC cells were identified in one spoiled beer sample based on MRS agar growth, AODC and Live/Dead BacLight bacterial viability kit methodologies with fluorescent microscopy and flow cytometer. Coupled with 16*S rRNA* gene sequencing, genomic sequencing results revealed that the culturable and VBNC cells from the spoiled beer sample were an individual *L. harbinensis* strain BM-LH14723.

VBNC state is considered a survival mechanism for bacteria under harsh environmental conditions. Bacteria in the VBNC maintain cell integrity and showed resistance against a wide variety of stress environments, including those commonly used in food preservation (e.g. low temperature, desiccation)^19^. As commonly used beer storage condition, low temperature is a typical harsh environmental condition beer spoilage bacteria encountered. Low-temperature or cold treatment combined with oligotrophic conditions have also been shown to be important to induce the VBNC formation of various bacteria in previous studies^8,14,15,20–31^. In this study, low temperature storage was able to induce *L. harbinensis* strain BM-LH14723 to enter into the VBNC state, which indicated beer spoilage *L. harbinensis* is capable of enter into the VBNC state during beer storage process. As beer spoilage bacteria were concerned, VBNC state of *L. lindneri* and *L. paracollinocides* have been induced by beer adaption coupled with sublethal heat treatment^5^. Also, our previous studies showed beer subculture and cold treatment were able to induce the VBNC state of *L. actotolerans*, *L. casei*, *L. plantarum*, and *L. lindneri*^9–11,19^. The induction of VBNC state *L. harbinensis* by beer subculturing suggested the ability of *L. harbinensis* to enter into the VBNC state during beer brewing process. In this study, both culturable and VBNC cells of an individual *L. harbinensis* strain BM-LH14723 were identified from spoiled beer, and its induction of VBNC state was determined by low-temperature storage in beer and beer subculturing. It confirmed the ability of *L. harbinensis* strain BM-LH14723 to enter into the VBNC state.

The VBNC state has been regard as a significant means of survival since the cells are able to increase metabolic activity and again become culturable^13^. It has been reported that the oxidative stress response in bacteria is associated with cold stress^32^, and addition of catalase could relieve oxidative stress and promote the recovery of nonculturable cells^23,33^. Consistently, the addition of catalase effectively resuscitated the VBNC *L. harbinensis* cells induced by both methods. It has been reported that transfer of cells to nutrient-rich environment initiates a metabolic imbalance, thus leading to a rapid production of superoxide and free radicals^34^. Furthermore, the antibacterial function of hop compounds has been reported to associate with efficient redox reactivity and cause cellular oxidative damage^35^. Therefore, it is possible that the VBNC *L. harbinensis* cells are stressed and sensitized to detoxify superoxide during active phenotypic adaptation to the beer environment containing high concentration of bitter hop compounds. Thus, the antioxidant capacity of catalase may alleviate these stress conditions.

The VBNC and resuscitated *L. harbinensis* cells remained viable and maintained similar beer-spoilage capacity to exponentially growing cells, which was somewhat in accordance with previous studies^13,22,30,36–43^. As beer brewery industry and *Lactobacillus* were concerned, the VBNC *L. paracollinocides*, *L. actotolerans*, *L. casei*, *L. plantarum*, and *L. lindneri* cells also exhibited beer-spoilage ability^5,9–11,22^. Nevertheless, with its detection in breweries and capacity to enter into VBNC state formation under low temperature in beer, *L. harbinensis* may represent a significant source of beer spoilage cases.

Concerning the VBNC state is a bacterial survival mechanism under stress conditions, genes associated stress response might play important roles in formation of the VBNC state. In the genome of *L. harbinensis* strain BM-LH14723, 6 universal stress proteins UspA, 3 predicted membrane GTPases involved in stress response, 1 putative stress-responsive transcriptional regulator, 2 stress-70 proteins, 1 general stress protein, 6 genes involved in response to oxidative stress, and 3 genes involved in response to stress were identified. Gene encoded oxidative stress response has been reported to related to cold stress response in bacteria^32^. The 6 genes involved in response to oxidative stress might be functional during the VBNC state formation process of *L. harbinensis* strain BM-LH14723 under low temperature condition. Sigma factor RpoS has been reported to play key role in enhancing stress resistance^44^ and associate with the VBNC state^45^. However, RpoS was absent in the genome of *L. harbinensis* strain BM-LH14723. Nevertheless, sigma factor RpoD and 6 other RNA polymerase sigma factors appeared in the genome might be alternative sigma factors involved in the formation of VBNC state of *L. harbinensis* strain BM-LH14723.

In this study, both culturable and VBNC cells of an individual *L. harbinensis* strain were identified from one spoiled beer. Based on the ability of *L. harbinensis* to enter into and resuscitated from the VBNC state, this study strongly suggests that the VBNC *L. harbinensis* cells in the beer sample likely contributed to this spoilage event. Moreover, addition of catalase to routine detection media may lead to more accurate and rapid diagnosis of spoilage possibility, prevent significant profit loss to the brewing industry, and improve food safety for consumers. The genomic analyses also improved our understanding on the intrinsic characteristics of *L. harbinensis* strain BM-LH14723 including the VBNC state associated genes. In summary, this study provides further evidence that entry into the VBNC state provides a unique survival strategy for LAB associated with beer spoilage.

## Materials and Methods

### Sample processing, strains and bacterial identification

In June 2014, one spoiled lager beer sample (pH 4.5, ethanol ≥ 3.6% v/v, bitterness units 7, 3 months after manufacturing with an expiration time of 12 months) was acquired in Guangzhou of South China. Following routine microorganism identification (plating the spoiled beer sample on MRS agar, observing the cells under microscope after filtering, and sequencing 16*S rRNA* gene upon PCR amplification and purification) and genomic sequencing using the bacterial genomic DNA extracted from the spoiled beer sample, one *L. harbinensis* strain was identified, which had been designated BM-LH14723. The culturable, total and viable cell numbers of *L. harbinensis* were calculated by MRS agar plates (Oxoid, UK), acridine orange direct count (AODC) method and Live/Dead BacLight bacterial viability kit (Molecular Probes, USA) with fluorescent microscope and flow cytometer, respectively^25,46^. The difference between culturable and viable number of *L. harbinensis* cells was also calculated to quantify the proportion of VBNC cells in spoiled beer.

### Whole genome sequencing

To validate the existent bacteria, the genomic DNA of bacterial cells from the spoiled beer sample and the isolated *L. harbinensis* cells were extracted by bacterial genomic DNA extraction kit (Sigma-Aldrich, USA) and subjected to genomic sequencing by Illumina HiSeq. 2500 platform and paired-end libraries, respectively^10,47^. Sequences were quality processed using the FastQC pipeline v.0.10.1 before assembly. Upon *de novo* assembly using Velvet v1.2.08, both genome sequences were aligned by progressive Mauve genome alignment software with default settings (http://darlinglab.org/mauve/mauve.html)^48^.

### Genome annotation

To determine the intrinsic characteristics of *L. harbinensis* strain BM-LH14723, genes were predicted through Glimmer 3.0^49^, RNAmmer (v1.2)^50^ and *tRNA*scan-SE (v1.21)^51^, respectively. Predicted genes were further annotated by online BLAST (http://blast.ncbi.nlm.nih.gov/Blast.cgi), KOBAS 2.0 (Xie *et al*., 2011) via KEGG pathway database^52^, COG functional classification system^53^, and local BLAST via NCBI-NR database and GO database^54^.

### Low-temperature storage in beer

Prior to VBNC state induction, the commercial beer were degassed for 10 min at 20 kHz at room temperature^19^. The low-temperature storage system was set up as follows. Approximately 10^7^ cells of *L. harbinensis* strain BM-LH14723 were inoculated and anaerobically subcultured at 26 °C in 10 mL of the degassed commercial beer. The exponentially growing cells were harvested by centrifugation at 5,000 rpm for 15 min at 4 °C, and then resuspended in 10 mL of degassed beer using 15-mL sterile polypropylene tube at a final density of 10^7^ cells/mL and maintained at 0 °C without shaking. A total of 50 low-temperature storage systems were prepared.

### Beer subculturing

Beer subculturing was performed as described by Suzuki *et al*. with some modification^5,9–11,19^. Approximately 10^7^ exponentially growing *L. harbinensis* BM-LH14723 cells were inoculated in degassed beer and anaerobically incubated at 26 °C. The exponentially growing cells were harvested by centrifugation at 5,000 rpm for 15 min at 4 °C, and then resuspended by 10 mL of degassed beer in 15-mL sterile polypropylene tube at a final density of 10^7^ cells/mL and anaerobically cultured at 26 °C. The interval of each subculture was 7 days.

### Enumeration of total, culturable, and viable cells

Total, culturable, and viable cells enumerations were performed every 7 days by taking out single tube of low-temperature storage system and before each subculture, respectively. The total cells number was determined by the AODC method with flow cytometer^47^. The number of culturable cells were accessed by a conventional plate culture procedure^18^. A hundred μL of subcultures was spread on MRS agar and incubated anaerobically at 26 °C. The inoculated agar plate was examined every day for colonies formation, and the day on which colonies were first observed was recorded as the time of positive detection. After 14 days of incubation, cells were counted to determine culturable cells number on agar plates. ‘Nonculturable’ was defined as the inability to grow on MRS agar until 14 days of incubation^9–11,19^. Cell viability was determined by using a Live/Dead BacLight bacterial viability kit (Molecular Probes, USA) with fluorescent microscopy and flow cytometer^12^.

### Propidium monoazide (PMA)-PCR verification

To verify the existence of VBNC cells, 0.5 μg/mL of PMA was added to 1 mL of the bacterial culture once the nonculturable cells identified. The culture was incubated on ice in dark for 10 min, subsequently exposed to halogen light with a distance of 15 cm for 5 min for covalent binding of PMA to DNA, and further cooled to room temperature after the reaction^55^. Genomic DNA was extracted using bacterial genomic DNA extraction kit (Sigma-Aldrich, USA) and used as a template to amplify the *16S rRNA* gene (5′-AGAGTTTGATCCTGGCTCAG-3′, 5′-CTACGGCTACCTTGTTACGA-3′) in PCR assay.

### Gradually temperature upshift

Upon entry into the VBNC state, 6 portions (100 μL each) of *L. harbinensis* cells were added into 1.5-mL sterile polypropylene tubes, respectively. Each sample was initially incubated at 10 °C for 1 h and then the incubation temperature was increased by 5 °C every 1 h until reaching 35 °C. One portion of cells was taken out every hour to determine culturability by plating on MRS agar and incubated anaerobically at 26 °C.

### Changing medium concentration

MRS agar plates with concentrations at 0%, 25%, 50%, 75%, 100%, 150%, and 200% were prepared to recover culturability of VBNC *L. harbinensis* cells. A hundred μL of VBNC cells were plating on different concentrations of MRS agar, respectively, and incubated anaerobically at 26 °C to observe colonies formation.

### Adding chemicals

To determine effects of some chemicals on resuscitation of the VBNC *L. harbinensis* cells, MRS agar plates with the addition of 10 μL Tween-20, 10 μL tween-80, 0.05 g vitamin C, 0.05 g vitamin B2, and 800 U catalase (Sigma-Aldrich, USA) were used, respectively. A hundred μL of VBNC cells were plating on MRS agar with the addition of different chemicals, respectively, and incubated anaerobically at 26 °C to observe colonies formation. In addition, different concentrations of catalase (0, 200, 500, 800, 1000, 1200, and 1500 U/plate) were added to MRS agar to resuscitate *L. harbinensis*. Simultaneously, heat-denatured catalase (60 °C for 15 min) served as a control.

### Evaluation of beer-spoilage ability

The beer-spoilage ability was investigated using established protocols for “growth in beer test”^4^. Approximately 10^5^ cells/mL of the strain in exponentially growing, VBNC, and resuscitated state were inoculated into 3 groups of commercial degassed lager beer (pH 4.5, ethanol ≥3.6% v/v, bitterness units 7) under sterile conditions at room temperature, respectively, with uninoculated beer as negative control. With beers incubated at 26 °C for up to 30 days, visible growth were examined every day and cellular culturability and viability were detected every 7 days. Subsequently lactic acid and acetic acid concentration were analyzed by reversed-phase high performance liquid chromatography (RP-HPLC). The diacetyl concentration was measured by Head Space Gas Chromatography. Lactic acid, acetic acid and diacetyl were quantified by the external standard method^9–11,19^.

### Statistical analysis

Data are presented as mean ± standard deviation (SD) of three independent biological replicates. Statistical comparisons were made by one-way analysis of variance followed by Tukey’s comparison test (XLstat software). A *p*-value < 0.05 was considered to be significant.

## Electronic supplementary material

Table S1

Table S2

Table S3

Table S4
